# Targeting Apoptosis Signalling Kinase-1 (ASK-1) Does Not Prevent the Development of Neuropathy in Streptozotocin-Induced Diabetic Mice

**DOI:** 10.1371/journal.pone.0107437

**Published:** 2014-10-16

**Authors:** Victoria L. Newton, Sumia Ali, Graham Duddy, Alan J. Whitmarsh, Natalie J. Gardiner

**Affiliations:** 1 Faculty of Life Sciences, University of Manchester, Manchester, United Kingdom; 2 Platform Technology and Sciences, GlaxoSmithKline, Stevenage, Herts, United Kingdom; Hirosaki University Graduate School of Medicine, Japan

## Abstract

Apoptosis signal-regulating kinase-1 (ASK1) is a mitogen-activated protein 3 kinase (MAPKKK/MAP3K) which lies upstream of the stress-activated MAPKs, JNK and p38. ASK1 may be activated by a variety of extracellular and intracellular stimuli. MAP kinase activation in the sensory nervous system as a result of diabetes has been shown in numerous preclinical and clinical studies. As a common upstream activator of both p38 and JNK, we hypothesised that activation of ASK1 contributes to nerve dysfunction in diabetic neuropathy. We therefore wanted to characterize the expression of ASK1 in sensory neurons, and determine whether the absence of functional ASK1 would protect against the development of neuropathy in a mouse model of experimental diabetes. ASK1 mRNA and protein is constitutively expressed by multiple populations of sensory neurons of the adult mouse lumbar DRG. Diabetes was induced in male C57BL/6 and transgenic ASK1 kinase-inactive (ASK1n) mice using streptozotocin. Levels of ASK1 do not change in the DRG, spinal cord, or sciatic nerve following induction of diabetes. However, levels of ASK2 mRNA increase in the spinal cord at 4 weeks of diabetes, which could represent a future target for this field. Neither motor nerve conduction velocity deficits, nor thermal or mechanical hypoalgesia were prevented or ameliorated in diabetic ASK1n mice. These results suggest that activation of ASK1 is not responsible for the nerve deficits observed in this mouse model of diabetic neuropathy.

## Introduction

A number of apoptosis-signalling kinase species have been identified: ASK1 [1], ASK2 [2] and ASK3 [3]. As their name suggests, activation of these mitogen-activated protein (MAP) 3 kinases can trigger apoptosis of the cell in response to cellular stress, for example: oxidative stress, tumour necrosis factor-α (TNF-α) and Fas antigen activation, all of which may be induced by disease [3]. ASK1 is mainly localised in the cytoplasm [4] although both ASK1 and ASK2 have also been detected within the nucleus and mitochondria [5]. ASK1 is a tightly regulated MAP 3 kinase, which lies upstream of JNK and p38 MAP kinases. Activation of ASK1 can lead to activation of both c-Jun N-terminal kinase (JNK) and p38 mitogen-activated kinase (p38) MAP kinase species [6].

Given the highly regulated nature of ASK1, its activity is tightly controlled by a number of regulatory molecules able to bind and phosphorylate ASK1 at specific sites [7]–[9]. Important ASK1 regulatory proteins are ASK2 and thioredoxin [10], [11]. ASK2 is a MAP 3 kinase not as widely studied, but closely related to ASK1 [2], and able to aid ASK1 activation when ASK1 and ASK2 form a heterodimer [11]. Thioredoxin binds with ASK1 in its reduced form, preventing phosphorylation of its major activation site. Dissociation of thioredoxin is required for ASK1 activation [12].

It has previously been reported that activation of MAP kinases, particularly p38, in experimental diabetes contributes towards the pathology of diabetic neuropathy. Treatment of dissociated adult neuronal cultures with glucose activates p38 MAP kinase, and also JNK [13]. Increases in p38 and JNK activation are observed in the sensory nervous system of rodents with diabetic neuropathy [14]–[16]. Administration of p38 inhibitors (SB 239063 and SB 203580) ameliorate symptoms of experimental diabetic neuropathy in rat models, both in terms of preventing NCV deficits [17], and reversing heightened mechanical sensitivity [16]. Importantly, increased levels of JNK and p38 have also been detected in sural nerve biopsies taken clinically from diabetic patients [13].

Since ASK1 is an upstream activator of both the p38 and JNK branches of the MAP kinase cascade [18], we hypothesised that ASK1 activation in diabetes may lead to downstream activation of p38, contributing towards the neuropathic phenotype in streptozotocin induced diabetic mice.

ASK1 transcription has previously been described in a range of non-neuronal tissues [1], [19], however, to the best of our knowledge, has not yet been investigated in the sensory nervous system. In the present study we examine the expression of ASK1 RNA and protein in the adult mouse sensory nervous system (spinal cord, dorsal root ganglia and sciatic nerve) and the regulation of expression by diabetes. ASK1 kinase inactive mice (ASK1n: in which ASK1 is rendered non-functional by a point mutation in the catalytic domain (at the ATP binding site within exon 15)) show reduced pain behaviour in a peripheral inflammatory model [20]. We used these mice to assess whether ASK1 had a functional role in altered mechanical and thermal sensitivity thresholds in experimental diabetic neuropathy, and therefore whether ASK1 would be a suitable future drug target in the treatment of diabetic neuropathy.

## Methods

### Animal details

All experiments were performed in accordance with institutional ethical regulations and authorized under the UK Animals (Scientific Procedures) Act (1986). Experiments were conducted using age-matched C57BL/6 (20–32 g; 6–12 weeks; Charles River, UK) and ASK1n (20–32 g; 6–12 weeks; University of Manchester, UK) male mice. ASK1n mice were produced by GlaxoSmithKline on a C57 background (GSK, UK) by a point mutation of the ASK1 gene within exon 15 at the ATP binding site (residue 716, lysine converted to an arginine)[20]. Mutation of this ATP binding site (K709 in human ASK1) is widely used to render ASK1 kinase inactive [21]–[24]. In brief, a targeting vector containing the mouse ASK1 gene, mutated within exon 15 at residue 716 (lysine converted to arginine) within the ATP binding site domain was produced using standard recombinant DNA techniques in *Escherichia coli* and homologous recombination in *Saccharomyces Cerevisiae*. This was then transfected into E14.1 embryonic stem cells and homologous recombination confirmed by Southern blot analysis. A single targeted clone was expanded and injected into C57 blastocysts. The resulting male chimaeras were bred with C57 wild-type females, the point mutation was confirmed to be germline transmitted and heterozygous mice for the mutation crossed to produce wild-type, heterozygous and homozygous mice. Homozygous mice were then inter-bred for use in phenotypic studies. Mice were genotyped (using the primers; ASK-1 Forward 5′GATCCCCTAAAGAAGCCCATC3′ and ASK-1 Reverse 5′ TGGTGTTTTGACTGGACAGC 3′) and the DNA amplicon was digested by Nhe-1 restriction enzyme and/or sequenced for the point mutation.

All mice were genotyped (using the primers; ASK-1 Forward 5′GATCCCCTAAAGAAGCCCATC3′, and ASK-1 Reverse 5′ TGGTGTTTTGACTGGACAGC 3′). The DNA amplicon was digested by Nhe-1 restriction enzyme and/or sequenced for the point mutation. Animals were maintained on a 12∶12 hour light:dark cycle at 21°C±2 and at 45% ±10 humidity. Animals had free access to standard rodent laboratory chow (Beekay International) and tap water. Animals were allocated to treatment groups using an online random number generator tool (RANDOM.ORG; http://www.random.org/) and also tested each day in a randomised order, again using RANDOM.ORG to minimise subjective bias.

### Kinase Activity Assay

The dominant negative phenotype of the mice was confirmed using an *in-vitro* kinase assay. ASK1 activity was directly measured using myelin basic protein (MBP) as the exogenous substrate. ASK1 was immunoprecipiated from the brains of ASK1n transgenics (*n* = 4) and age matched controls (*n* = 4). Briefly, brains were homogenised in triton-X lysis buffer (TLB) containing 20 mM Tris-HCL (pH 7.4), 137 mM NaCl, 25 mM sodium β-glycerophosphate, 2 mM sodium pyrophosphate, 2 mM EDTA, 1 mM sodium orthovanadate, 10% glycerol, 1% Triton X-100, plus protease inhibitors; 1 mM PMSF and 5 µg/ml each of leupeptin and aprotinin (Sigma Aldrich, UK). The homogenates were centrifuged three times for 10 minutes at 15000×g at 4°C. Brain lysates (400 µL) were incubated on a rotating platform with 2 µg of anti-ASK-1 specific antibody (sc7932, Santa Cruz Biotechnology, USA) or 2 µg mIgG antibody isotype control (sc2025, Santa Cruz Biotechnology, USA) for 3 hours, followed by a further incubation with 20 µL of protein A Sepharose beads (Sigma Aldrich, UK) for 30 minutes, at 4°C. Coated beads were harvested using a magnet, washed three times in 1 ml TLB and twice in 1 ml of kinase buffer (KB: 25 mM Hepes (pH 7.4), 25 mM sodium β-glycerophosphate, 25 mM MgCl_2_, 0.1 mM sodium orthovandate and 0.5 mM dithiothreitol). The kinase reaction was carried out by mixing the beads from each sample with 30 µl reaction mix containing; 27 L KB, 2 mL MBP (5 µg), 1 µL 1 mM ATP, 0.5 µl 10 mCi/mL [γ-^32^P]ATP(6000 Ci/mmol) for 30 minutes at 30°C. The kinase reaction was terminated by the addition of 10 µL of SDS gel loading buffer and boiling at 100°C for 5 minutes. The supernatants containing both MBP and ASK1 were electrophoresed on a 12% SDS-PAGE mini gel for 1 hour at 150 V. The gel was stained for 10 minutes with coomassie stain to visualize the substrate and then destained until the gel background became clear. The gels were then dried and subjected to autoradiography using Kodak X-Omat film to measure the incorporation of ^32^P from [γ-^32^P]ATP into MBP, the intensity of each band was scanned and analysed with Sigmascan software. The band intensity for mIgG immunoprecipitate and MBP reaction was used to indicate background kinase activity and was subtracted from all other bands.

### Induction of diabetes and drug delivery

Diabetes was induced using a 180 mg/kg dose of streptozotocin (STZ; Sigma Aldrich, UK) injected intraperitoneally following a 12-hour fast. STZ was dissolved in sterile saline immediately prior to injection.

Hyperglycaemia was confirmed 6 days after STZ administration with blood collected from a tail vein, which was measured for glucose content using a strip-operated reflectance photometer (OptimumPlus; MediSense, UK or ACCU-CHEK Aviva; Roche, Germany). Animals were deemed diabetic at blood glucose levels of 15 mmol/L and above, and maintained up to 12 weeks after the confirmation of hyperglycaemia.

### Behavioural testing

To assess mechanical sensitivity, animals were acclimatised to an elevated, wire-bottomed, compartmentalised testing chamber (Ugo Basile, Italy). A 2 g Von Frey filament was then manually applied alternately to the left and right hindpaws 6 times in total (3 on each foot) and the percentage foot withdrawal response calculated.

For thermal testing, animals were acclimatised to a Perspex-bottomed testing chamber (Ugo Basile, Italy). The time taken for the animal to remove its hindpaw from the infra-red heat stimulus on the Hargreaves apparatus (Ugo Basile, Italy) was automatically recorded for each foot 2 times, with repeat testing on the same foot being no less than 5 minutes apart. A time and intensity threshold was in place to prevent any tissue damage.

In all cases, testing was repeated on consecutive days. For baseline measurements, recordings were taken following a day of acclimatisation, where animals were placed in the cages, but were not tested. All behaviour scores were obtained from all values obtained over 2–3 days of testing.

### Measurement of nerve conduction velocity (NCV)

Mice were terminally anaesthetised with isoflurane (4% in oxygen, maintained at 2%) and maintained on a warmed blanket. A fine stimulating electrode was placed percutaneously into the sciatic notch and another placed subcutaneously. Graded electrical stimuli (1–10 mA) were applied as 0.1 ms square pulses of varying amplitude using a Neurolog stimulus isolator, pulse generator and amplifier. The resulting evoked electromyograms (m-waves) were recorded from the interossei muscles of the foot using ABI Scope version 3.6.8 for Powerlab 4 software (ADInstruments, Australia). The sciatic notch electrode was then moved to near the Achilles tendon and the stimuli repeated., Near-nerve temperature was monitored at end of NCV assessment using a small thermistor. The distance between the two stimulating sites was measured and the time difference in m-wave latency between the two sites used to calculate the motor NCV.

### Immunohistochemistry

Animals were culled by anaesthetic overdose and tissue was rapidly dissected and post-fixed in ice-cold 4% paraformaldehyde for 4 hours. Tissue was cryoprotected at 2–8°C in 10% sucrose in 0.1 M phosphate buffer for 18–24 hours followed by 30% sucrose in 0.1 M phosphate buffer for a further 18–24 hours. Tissue was then embedded in OCT embedding matrix media (Thermo Shandon Ltd, UK) and frozen on dry ice. Transverse sections were cryostat-cut (12 µm) and thaw-mounted onto Superfrost Plus slides (Fisher Scientific, UK).

For staining of ASK1, antigen retrieval was first performed by boiling sections in sodium citrate buffer (0.01 M tri-sodium citrate, 0.05% v/v tween 20, pH 6). Slides were then washed in phosphate buffered saline (PBS) before non-specific binding was blocked for an hour (10% donkey serum in 0.2% triton-X in PBS at room temperature) and sections then incubated for 48 hours with primary rabbit anti-ASK1 antibody (1∶250; Abcam, UK; 5% donkey serum in 0.2% triton-X PBS, 2–8°C); mouse anti-calcitonin gene related peptide (CGRP; 1∶500; Sigma) and fluorescein labeled isolectin B4 (IB4; 30 µg/ml; Vector labs, UK). After washing, primary antibody was visualised using appropriate cyanine 3 (Cy3)-conjugated (1∶400; Jackson ImmunoResearch, USA) and Alexa Fluor 647(1∶750; Invitrogen, UK) antibodies for 2 hours at room temperature (10% donkey serum, in 0.2% triton-X in PBS).

Negative control sections were produced with each batch of immunostaining with the absence of primary antibody. Staining was visualised on Leica DMR microscope and images captured using a Hamamatsu digital C4742-95 digital camera or a BX51 upright microscope and Coolsnap EZ camera. Sensory neuron profiles from at least 4 randomly selected sections of L4/5 DRG from 4 animals were traced using an image analysis program (Sigma Scan Pro Software), and the intensity of ASK1-immunoreactivity determined for each neuron. ‘High-levels’ of immunoreactivity were assessed by calculating the mean intensity from 4 neurons deemed ‘highly-immunoreactive’, and this threshold used to calculate the percentage of neurons with high levels of ASK1.

### Western blotting

Tissue was rapidly dissected and immediately frozen on dry-ice. Tissue samples were later homogenised in ice-cold lysis buffer (25 mM Tris HCl pH 7.4, 15 mM NaCl, 10 mM NaF, 10 mM Na pyrophosphate, 2 mM EDTA, 0.2 mM Na_4_OV_3_, 1 mM PMSF, Protease Inhibitor Cocktail (1∶200; Sigma Aldrich), 1% (v/v) triton-X) using the FastPrep bead beater system (MP Biomedicals, USA). Proteins were resolved through an 8% or 10% SDS-polyacrylamide gels and transferred to nitrocellulose. Non-specific binding was reduced by incubation of membranes in 10% skimmed milk (Marvel, UK) dissolved in tween buffer (150 mM NaCl, 10 mM Tris HCL, 0.05% (v/v) Tween 20, pH 7.6) and then incubated overnight at 2–8°C in primary rabbit anti-ASK1 (1∶2000: Abcam, UK; in 5% milk in tween buffer; 50 µg of spinal cord and sciatic nerve and 20 µg of DRG protein lysate per lane) or rabbit anti-phosphorylated/total p38 (1∶500: Cell Signaling, UK; in tween buffer; 20 µg of spinal cord lysate or 25 µg DRG lysate loaded per lane). After washing, membranes were incubated for 1 hour in secondary horseradish peroxidase (HRP)-linked anti-rabbit IgG antibody (1∶1000 dilution; Dako, Denmark; in 5% milk in tween buffer). Membranes were washed before visualisation of bound antibody using Amersham ECL Plus kit (GE Healthcare, UK). The films were scanned, and the total band intensity for each sample was calculated using SigmaScan Pro5 software (SPSS, Chicago, IL). ERK1/p44 band intensities were used to normalise protein loading. An ASK1 protein control was generated by transfection of cos7 cells with human ASK1 construct (gift; A. Whitmarsh, University of Manchester). Transfection was carried out using the jetPEI system according to manufacturer’s instructions (Polyplus, France). Cells were scraped into an Eppendorf tube and left to lyse on ice for 10 minutes. Lysates were then spun for 10 minutes at 9660 g at 4°C and the supernatant containing ASK1 removed and treated as for tissue lysates.

### Real-time PCR

Tissue was rapidly dissected into RNA later (Ambion, UK), stored initially overnight at 4°C and then at −80°C. Samples were removed from RNA later, placed in 1 mL of Trizol reagent (Qiagen, UK) and homogenised using the FastPrep bead beater system (MP Biomedicals, USA). RNA extraction then proceeded as per the Qiagen RNeasy Lipid Tissue Kit manufacturer’s instructions (Qiagen, UK). Any contaminating DNA was removed by incubation of the eluted RNA solution at 37°C with DNAse I for 30 minutes using a DNA*-*free Kit as per manufacturer’s instructions (Ambion, UK). RNA concentration was determined using a NanoDrop 8000 (Nanodrop, USA, software version 3.1.1).

RNA was then converted to cDNA using a Taqman Reverse Transcription Reagent Kit (1000 ng RNA; Applied Biosystems, UK). The reaction was performed at 25°C for 10 minutes followed by 30 minutes at 48°C before deactivation of the reverse transcriptase at 95°C for 5 minutes. A negative control containing no RNA template was included with each batch of samples processed and then used in a subsequent PCR reaction to confirm that there was no contamination of cDNA.

SYBR green with ROX reference dye was used to quantify cDNA transcripts (SYBR Green PCR Master Mix; Applied Biosystems, UK). ASK1 primers were used at 10 pmol, ASK2 and cyclophilin B at 5 pmol and thioredoxin at 2.5 pmol:

(ASK1: Forward-TGCCAGAAGAATACTGTGTGC, Reverse- TACTGGCTGGAACTCGCTTG; ASK2: Forward-CGGAGACTTTCACAGGACT, Reverse-TTGTACATGCCCACCTGAAA; Thioredoxin: Forward-GTCGTGGTGGACTTCTCTGCTA, Reverse-TTGTCACAGAGGGAATGGAAGA).

Samples were run in triplicate against cyclophilin B housekeeping gene (Forward- TAGAGGGCATGGATGTGGTAC, Reverse- GCCGGAGTCGACAAGATG), which was also performed in triplicate, all at 50°C for 2 minutes, 95°C for 10 minutes, followed by 40 cycles of 95°C for 15 seconds and 1 minute at 60°C. A dissociation reaction was performed following each run to ensure only a single product had been formed. A no-template negative control was also performed in triplicate for each primer on each run. All reactions were undertaken using an ABIPrism 7700 sequence detector system (Applied Bioscience, UK).

All primers used encompassed sequences from different exons to prevent amplification of any genomic DNA which may have evaded degradation. PCR products for each primer were run also run on a 2% Tris-acetate-EDTA (TAE; X10 concentrate; Sigma Aldrich, UK) agarose gel containing ethidium bromide (0.8 µg/mL of agarose) to further ensure only a single product had been produced. PCR products from each primer set were also sequenced to ensure the correct cDNA of interest was being amplified.

Triplicate CT values obtained for each sample were averaged and the gene of interest normalised to the cyclophilin B control. The 2^−Δ*C*^_T_ was then calculated and group results statistically analysed in an unpaired Student’s *t*-test.

### Lipid peroxidation ELISA

A 4-hydroxynonenal (HNE) his-adduct ELISA (Cell Biolabs inc, STA-334) was used to assess lipid peroxidation in 20 µg/mL of homogenised spinal cord protein samples according to the manufacturer’s instructions.

### Statistical analysis

A Student’s unpaired *t*-test or Mann-Whitney U was used to compare control and diabetic samples or C57 and ASK1n diabetic animals where appropriate. A 2-way ANOVA was used to analyse all experiments where C57 and ASK1n genotypes were compared.

## Results

### ASK1 is expressed in the adult mouse sensory nervous system

**Figure 1:**
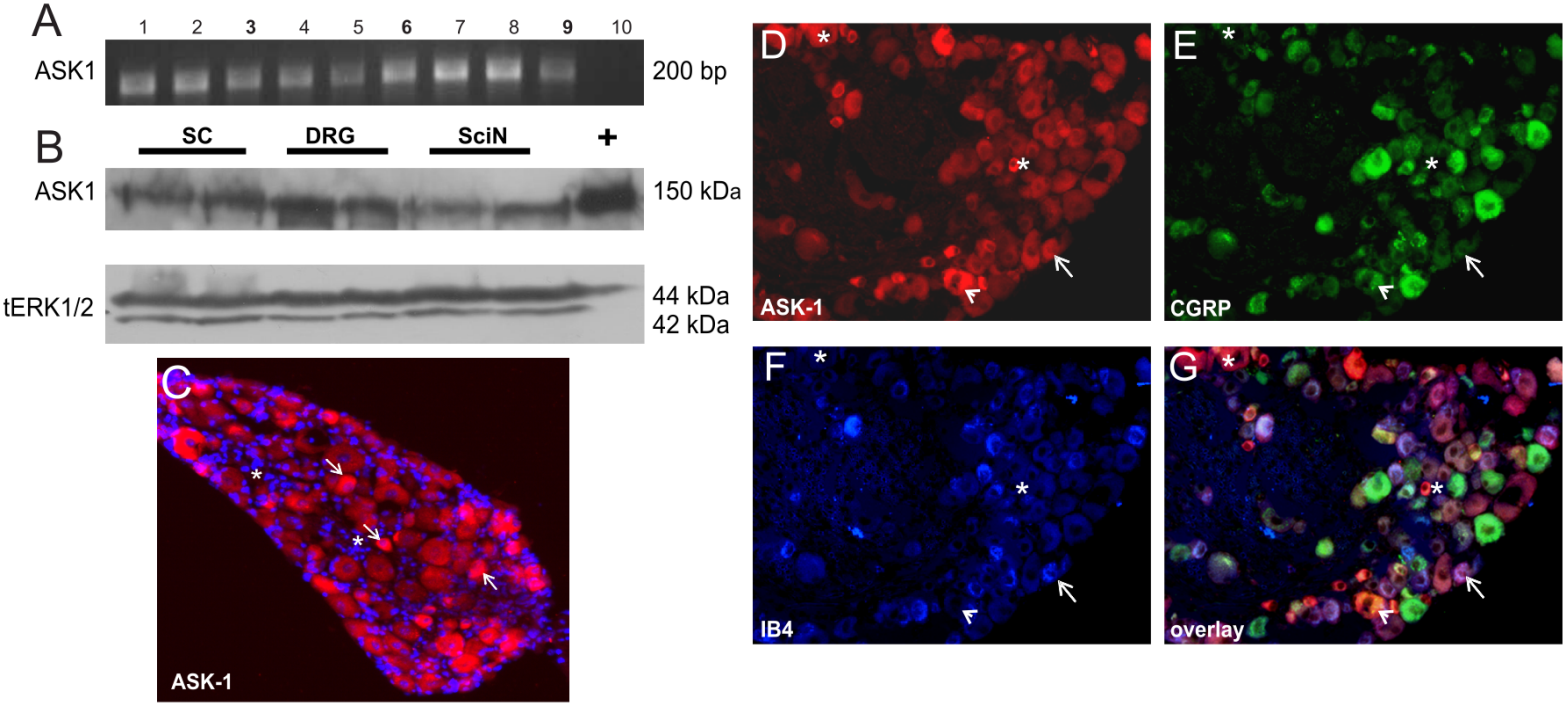
ASK1 can be detected in the sensory nervous system. (A) A 215 bp ASK1 amplicon can be amplified from RNA obtained from control male C57 mouse tissues: 1.liver; 2.heart; 3.spinal cord (SC); 4.skeletal muscle; 5.pancreas; 6.DRG; 7.kidney; 8.lung; 9.sciatic nerve (SciN); 10.negative control (water; representative image shown from n = 3). (B) ASK1 protein is present in SC, DRG and SciN, total ERK is shown as a loading control and (+) ASK1 recombinantly expressed in cos7 cells as a positive control. (C,D) Representative photomicrographs of ASK1-ir (red) in L4/5 DRG. (C) DAPI nuclear staining is shown in blue. Triple labelling on sections with (D) anti-ASK-1; anti-CGRP (E, arrowheads) and IB4 (F, arrow) indicates that a population of neurons with high ASK1-ir do not express CGRP-ir or bind IB4 (asterisks, D–G). Scale bar: 50 µm.

Both the ASK1 mRNA transcript (Fig. 1A) and ASK1 protein (Fig. 1B) could be detected from a range of mouse sensory tissues including the sciatic nerve, lumbar (L) 4/5 dorsal root ganglia (DRG) and L4/5 spinal cord. Immunohistochemical analysis revealed that ASK1-immunoreactivity (-ir) is expressed in the soma of all sensory neurons (arrows, Fig. 1C, D) but not the satellite glial cells (asterisks; Fig. 1C) in L4/5 DRG. A small population of sensory neurons expressed relatively high levels of ASK1-ir (5.9±1.6% of total neuronal profiles expressed high ASK1-ir (in the 4 week cohort of mice), and 13.6±3.6% (in the older 12 week cohort); *n* = 4 mice per condition). To phenotypically characterize this population we performed triple fluorescence-labelling experiments with anti-ASK1, anti-calcitonin gene-related peptide (CGRP) to label small-medium diameter peptidergic neurons and FITC-conjugated isolectin B4 (IB4), a marker for non-peptidergic small-diameter sensory neurons. ASK1-ir colocalised with both CGRP (arrowheads, Fig. 1 D,E,G) and IB4 (arrows, Fig. 1D,F,G), and was also expressed in populations of large- and small- (CGRP and IB4-negative) diameter neurons (asterisks, Fig. 1D, G). Omission of primary antisera resulted in loss of ASK1-ir (data not shown). Therefore, ASK1 is expressed, at variable levels, by multiple populations of sensory neurons in the mouse DRG.

### ASK1 levels do not change in the spinal cord and DRG in experimental diabetes

We wanted to next determine whether expression of ASK1 changed as a result of streptozotocin-induced diabetes. We focused on two timepoints of diabetes: 4 weeks, to look for early changes, and a 12-week time point with an established neuropathic phenotype. Diabetic mice at both timepoints were significantly lighter and hyperglycaemic compared with their age-matched non-diabetic counterparts (Table 1A,B).

**Table 1:** Blood glucose and body weight data.

Cohort	Timepoint (weeks)	Genotype *(n number)*	Body Weight (g)		Terminal Blood Glucose (mmol/L)	
			control	diabetic	control	diabetic
**A**	**4**	C57 *(10)*	26.0±1.8	22.7±2***	11.3±4.8	26.8±2.7***
	**12**	C57 *(10&11)*	29.2±1.8	22.7±1.8***	12.3±1.6	27.0±1.7***
**B**	**4**	C57 *(5&6)*	29.8±1.2	21.5±1.8***	10.0±2.4	27.8±0.0***
	**12**	C57 *(5&8)*	33.7±1.4	25.3±1.6***	10.2±1.5	26.9±1.7***
**C**	**0**	C57	26.0±1.5	25.4±1.6	NT	NT
		ASK1n	26.8±2.0	25.4±4.1	NT	NT
	**4**	C57 (10)	30.4±1.3	24.4±0.9***	8.1±1.6	26.1±2.2***
		ASK1n (11&12)	30.9±3.8	21.9±1.7***	8.04±1.1	23.2±5.0***
**D**	**0**	C57	25.8±2.0	25.1±1.7	NT	NT
		ASK1n	24.8±2.4	25.4±2.9	NT	NT
	**8**	C57 (10)	30.0±3.9	24.0±2.0***	8.0±1.5	21.1±6.3***
		ASK1n (12&14)	30.4±2.2	24.6±3.1***	7.2±1.8	23.1±4.4***
**E**	**0**	C57	27.5±1.6	27.2±1.9	NT	NT
		ASK1n	26.9±2.3^$^	28.1±2.6^$^	NT	NT
	**12**	C57 *(10&13)*	32.4±1.4	27.0±1.9***	9.9±3.5	24.0±4.9***
		ASK1n *(9&12)*	33.5±2.3	25.0±1.3***^#^	9.1±1.4	24.0±3.8***
**F**	**0**	C57	27.4±3.6	26.3±3.0	NT	NT
		ASK1n	29.2±1.8	28.7±2.6	NT	NT
	**4**	C57 *(9&11)*	30.9±3.8	23.9±2.9***	9.06±1.4	32.0±0.0***
		ASK1n *(9)*	31.2±1.8	27.0±3.0***	9.40±1.5	31.2±1.9***

Diabetic mice were hyperglycaemic and significantly lighter than controls. Tissue from control and diabetic C57 mice was used for initial Western blot/immunocytochemistry (A) and RNA extraction (B) after 4 and 12 weeks of diabetes. (C–D) The 4 and 8-week study of C57 and ASK1n mice showed that diabetic mice from both genotypes possessed similar blood glucose and showed equivalent weight loss. (E) While hyperglycaemia was equivalent in the 12-week cohort, diabetic ASK1n mice were significantly lighter at the end of the study than diabetic C57 mice (#p<0.05 in a 2-way ANOVA with Bonferroni *post hoc* tests). (F) Terminal hyperglycaemia and body weight was equivalent in the C57 and ASK1n mice used in a repeat behavioural study, however there was a small difference in start body weights between the genotypes (^$^p<0.05 overall in a 2-way ANOVA, but not significant in *post hoc* tests). The limit of detection on the blood glucose meter was 27.8 mmol/L. ***p<0.001 compared with control mice in a 2-way ANOVA with Bonferroni *post hoc* tests. Data are expressed as mean ± SD.

**Figure 2:**
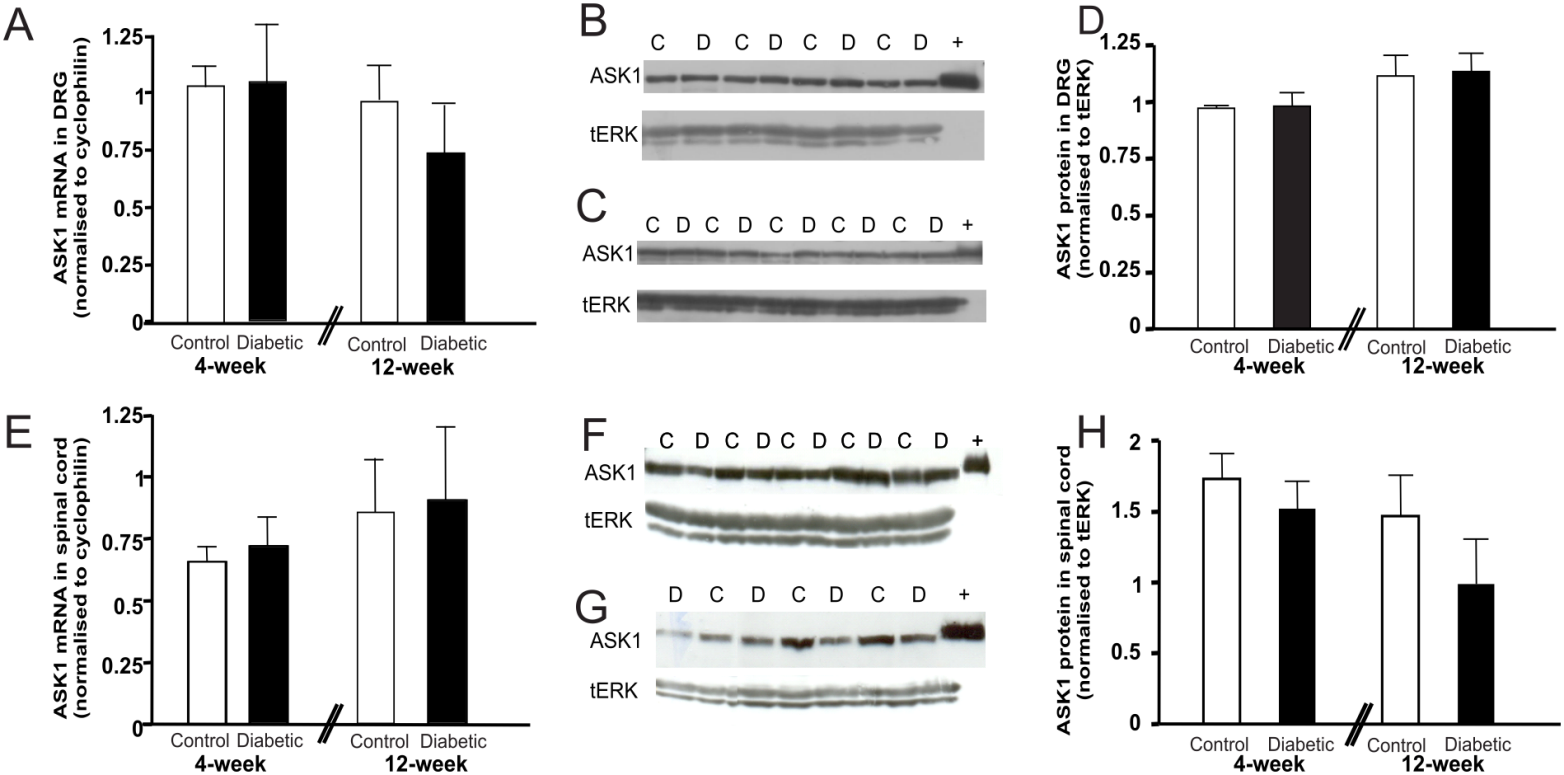
ASK1 levels in the DRG and spinal cord do not change in diabetes. Streptozotocin-induced diabetes did not cause any changes in levels of ASK1 mRNA in the DRG (A) or lumbar spinal cord (E) when normalised to cyclophilin B (4 or 12 weeks diabetes, p>0.05 Student’s unpaired *t*-test; *n* = 5–6). Representative Western blots of ASK1 and total ERK levels in DRG (B,C) and spinal cord (F,G) of age-matched control mice and mice at 4 and 12 weeks of diabetes (‘C’ control, ‘D’ diabetic and ‘+’ ASK1 control). (D, H) Densitometric analysis reveals no significant difference in ASK1 expression at the protein level between tissue from control and diabetic C57 mice at these timepoints. Densitometric analysis of ASK1 was normalised to ERK1 and compared using Student’s unpaired *t*-test. Data are expressed as mean intensity + SD; *n* = 4–6.

The levels of ASK1 mRNA (Fig. 2A) and protein (Fig. 2B–D) did not change in the DRG following 4 or 12 weeks of diabetes compared with controls (p>0.05). Similarly, there was no significant change in the proportion of neurons with high ASK1-ir in L4/5 DRG from diabetic mice (6.7±3.3% (4 week cohort) and 9.7±3.3% (12 week cohort) of total neuronal profiles expressed high ASK1-ir, data not shown). ASK1 expression was also examined in the spinal cord, again there were no differences in the levels of ASK1 mRNA (Fig. 2E) or protein (Fig. 2F–H) between control and diabetic mice.

Therefore, ASK1 expression does not change in the spinal cord or DRG as a result of diabetes, at either timepoint.

### ASK2 is increased in the spinal cord of diabetic mice

**Figure 3:**
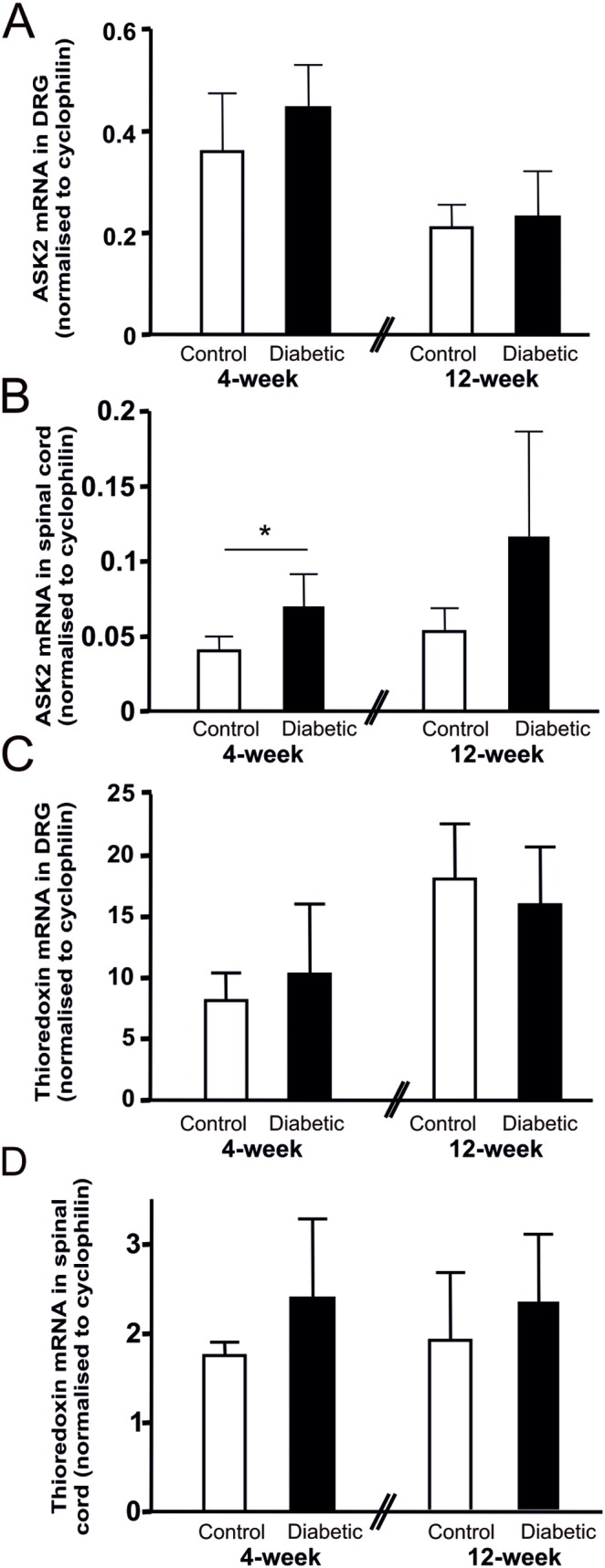
ASK2 mRNA levels increase in the spinal cord of 4-week diabetic mice compared with controls. (A) ASK2 mRNA levels do not change in the DRG in diabetes, however there is a significant increase in ASK2 in spinal cords from 4-week diabetic mice compared with controls (B, *p<0.05; Student’s unpaired *t*-test). (C,D) Thioredoxin mRNA levels in DRG and lumbar spinal cord did not change significantly in diabetes. All values were normalised to cyclophilin B. Data are expressed as mean + SD; n = 5–6.

Since ASK2 and thioredoxin are important regulatory proteins of ASK1, we investigated their expression levels in the DRG (Fig. 3A,C) and spinal cord (Fig. 3B,D) of diabetic and age-matched control mice. Whilst there was no significant change in ASK2 mRNA in the DRG (Fig. 3A), there was a significant increase in ASK2 in spinal cord samples after 4 weeks of diabetes, (Fig. 3B, p<0.05). In contrast, thioredoxin mRNA levels did not change in either tissue at these timepoints of diabetes (Fig. 3C,D).

### Characterization of ASK1n transgenic mice

**Figure 4:**
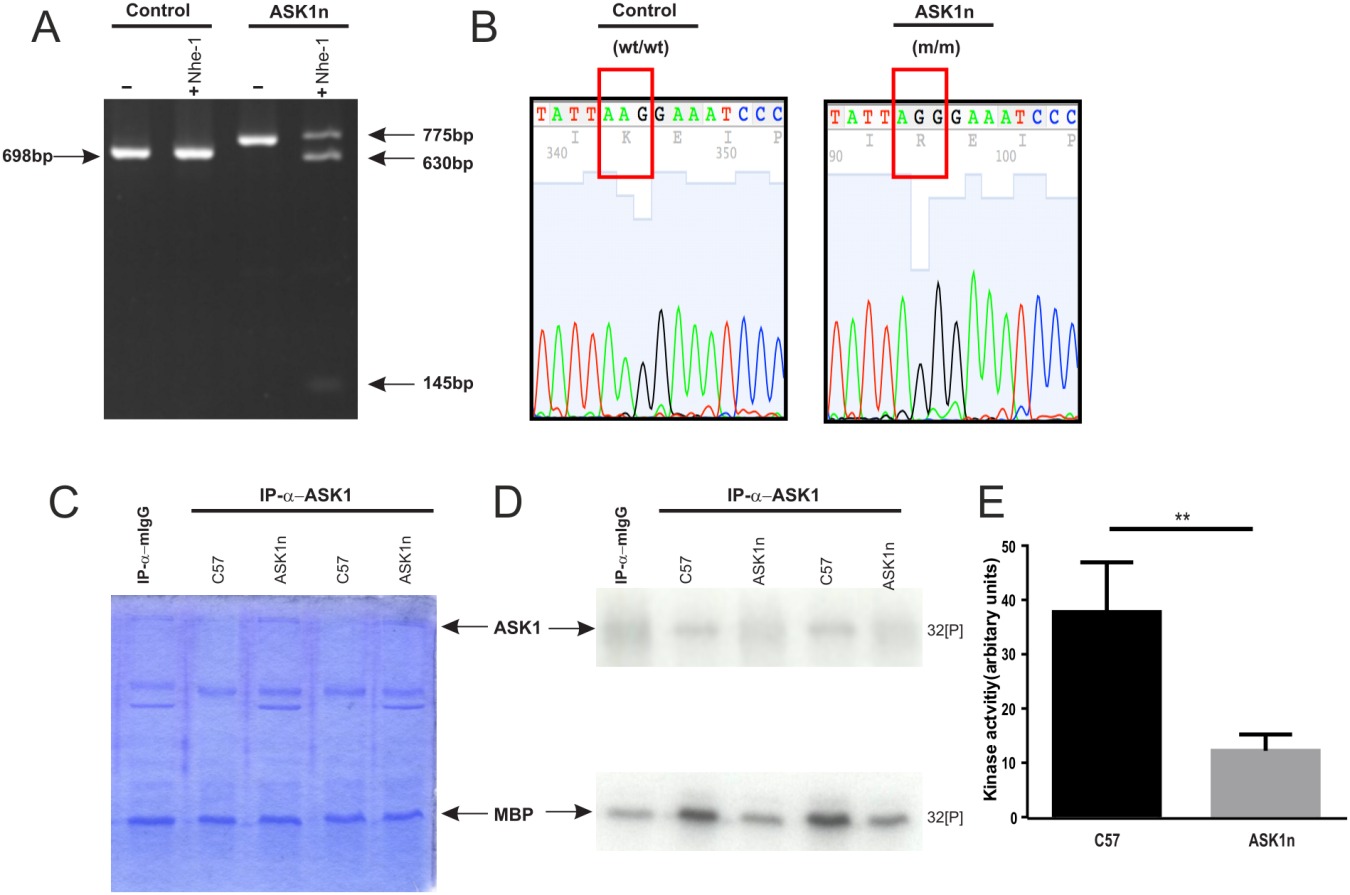
Characterisation of ASK1n transgenic mice. (A) The ASK1 sequence amplified from control mice cannot be digested with Nhe-1, whilst the sequence amplified from ASK1n can, confirming the presence of the modified ASK1 locus and the introduced Nhe-1 restriction site. (B) Chromatograph of the sequence flanking the mutation site of ASK1n mice, showing single G base substitution in homozygous mice and conversion of lysine (K) in control mice to arginine (R) in ASK1n mice. (C) Coomassie staining demonstrates that equal amounts of MBP substrate were added to each kinase reaction. (D) An immune complex kinase assay confirms phenotypic loss of kinase activity in brain tissue from ASK1n mice compared to control C57 mice (D, E **p<0.001; Students unpaired *t*-test).

We confirmed the genotype of transgenic ASK1 kinase inactive (ASK1n) mice (Fig. 4A,B) and the ASK1n phenotype using an in-vitro immune-complex kinase assay (Fig. 4C–E). ASK1 activity was directly measured using myelin basic protein (MBP) as an exogenous substrate (Fig. 4C). ASK1 kinase immunoprecipitated from brains of control C57 mice was able to phosphorylate MBP (Fig. 4D), whilst ASK1 immunoprecipitated from the brains of ASK1n mice was not, and had kinase activity similar to that observed when control brain lysate was immunoprecipitated with an antibody isotype control (anti-mIgG; Fig. 4C,D). Densitometric quantification confirmed this reduction in kinase activity (Fig. 4E; control kinase activity: 37.8±9.1 arbitrary units vs ASK1n kinase activity: 12.2±3.0 arbitary units, p<0.01).

### Induction of diabetes using streptozotocin is possible in the absence of functional ASK1

We were able to induce diabetes in adult male ASK1n mice using streptozotocin, and levels of both basal blood glucose and diabetes-associated hyperglycaemia were equivalent in C57 and ASK1n mice (Table 1). The 4- and 8-week cohorts of mice did not show any significant difference in body weight between genotypes (Table 1C,D). However, the diabetic ASK1n mice in the 12-week cohort, were significantly lighter than their diabetic C57 counterparts (Table 1E; p<0.05).

### p38 is activated in sciatic nerve from C57 mice with streptozotocin-induced diabetes

**Figure 5:**
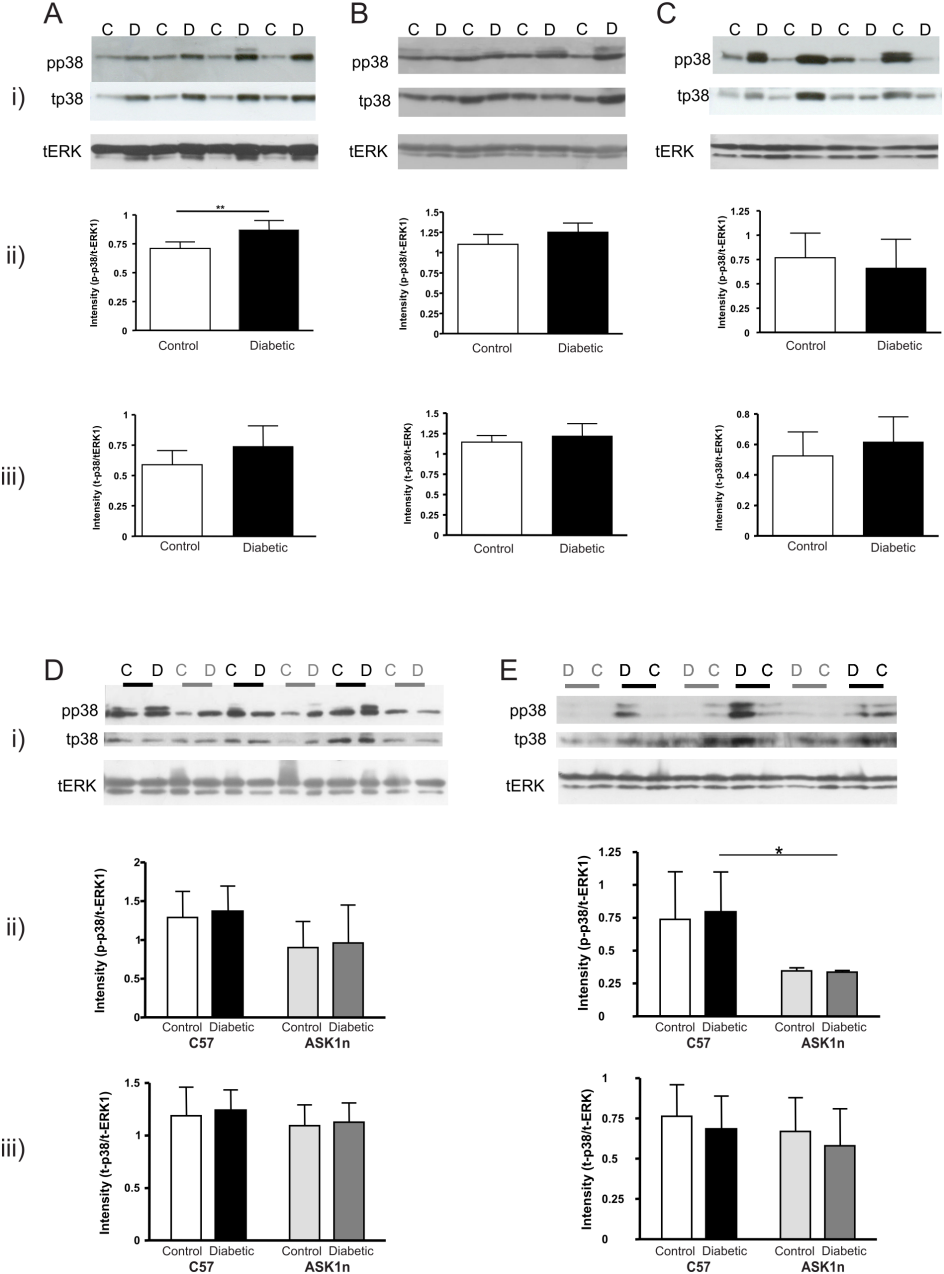
Phospho-p38 increases in the sciatic nerve from 4-week diabetic C57 mice. Representative images of levels of phospho (pp38), total p38 (tp38;) and total ERK (tERK) are displayed in i) from (A) sciatic nerve (B) DRG and (C) spinal cord protein samples from control and diabetic C57 mice (4-week). (A–C) Densitometric analysis of bands are presented as mean pp38 intensity relative to ERK1 (ii) and tp38 band intensity relative to ERK1 (iii). There was increased pp38 in sciatic nerve from 4-week diabetic mice compared with controls (**p<0.01 in a Student’s *t*-test, *n* = 5–6) and no difference in total p38. However, there was no significant difference in pp38 in DRG or spinal cord between control and diabetic mice at 4 weeks (p>0.05; Student’s unpaired *t*-tests. *n* = 5–6). There was no difference in pp38 or tp38 in sciatic nerve, DRG or spinal cord from 12-week diabetic compared with control C57 mice (data, not shown for 12 weeks). Western blots probed for pp38, tp38 and ERK (i) of sciatic nerve (D) and spinal cord (E) from 12-week C57 and ASK1n diabetic and control mice, with densitometric analysis of pp38 (ii) and tp38 (iii) relative to ERK1, confirmed that p38 activation is unchanged in nerve tissue from 12-week diabetic mice, and that overall, p38 activation is significantly reduced in ASK1n mice compared with C57 mice (p<0.05 in sciatic nerve; p<0.01 in spinal cord overall in a 2-way ANOVA; *p<0.05 in Bonferroni *post hoc* tests, *n* = 5–6). Data represents mean intensities + SD.

At 4 weeks of diabetes there was an increase in phosphorylated, but not total p38 levels in sciatic nerve from C57 diabetic mice compared with control mice (Fig. 5A). At 4 weeks there were no significant changes in p38 levels in the DRG (Fig. 5B) or spinal cord (Fig. 5C). Whilst, there was no significant difference in the levels of phosphorylated p38 at 12 weeks between control and diabetic mice of the same genotype, overall p38 activation levels were lower in sciatic nerve and spinal cord of ASK1n mice compared to C57 mice (Fig. 5D,E). Therefore ASK1n mice show reduced downstream p38 activation.

**Figure 6:**
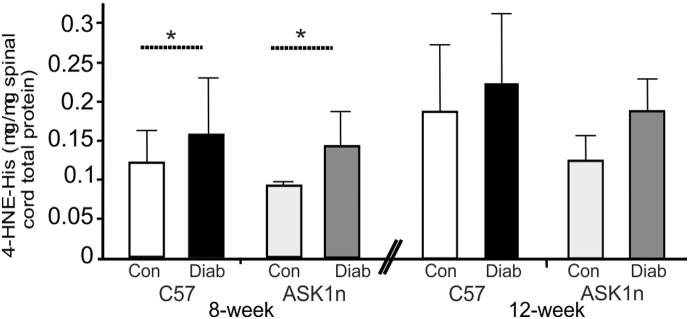
Levels of 4-HNE, a marker of oxidative stress, increase in the spinal cord of diabetic mice, this is not prevented or ameliorated by lack of functioning ASK1. 4-HNE levels (a secondary product of lipid peroxidation) in spinal cord protein samples, are higher in diabetic mice than in spinal cords obtained from control mice (C57 and ASK1n mice after 8 and 12 weeks of diabetes; *p<0.05, overall in a 2-way ANOVA, although not significantly different in *post hoc* tests *n* = 5). Data are expressed as mean values + SD.

Since increased oxidative stress is associated with diabetes [25], and also with activation of ASK1, we investigated the levels of 4-hydroxynonenal (4-HNE, a secondary product of lipid peroxidation) in the spinal cord. Levels of 4-HNE in spinal proteins from 8-week diabetic mice (Fig. 6; C57: 0.16 µg/µg±0.07 v.s. ASK1n: 0.15 µg/µg±0.04) were significantly greater than control levels (C57: 0.12 µg/µg±0.04 v.s. ASK1n: 0.09 µg/µg±0.01; p<0.05, overall in a 2-way ANOVA). Spinal 4-HNE levels of the 12-week cohort of mice showed a similar trend, although the difference between control and diabetic mice did not reach significance (Fig. 6). Whilst data shows a trend towards a lower baseline level of lipid peroxidation in ASK1n mice compared with C57s, this was not significant.

### Lack of functional ASK1 does not alleviate or prevent diabetes-associated mechanical and thermal hypoalgesia or nerve conduction deficits

**Figure 7:**
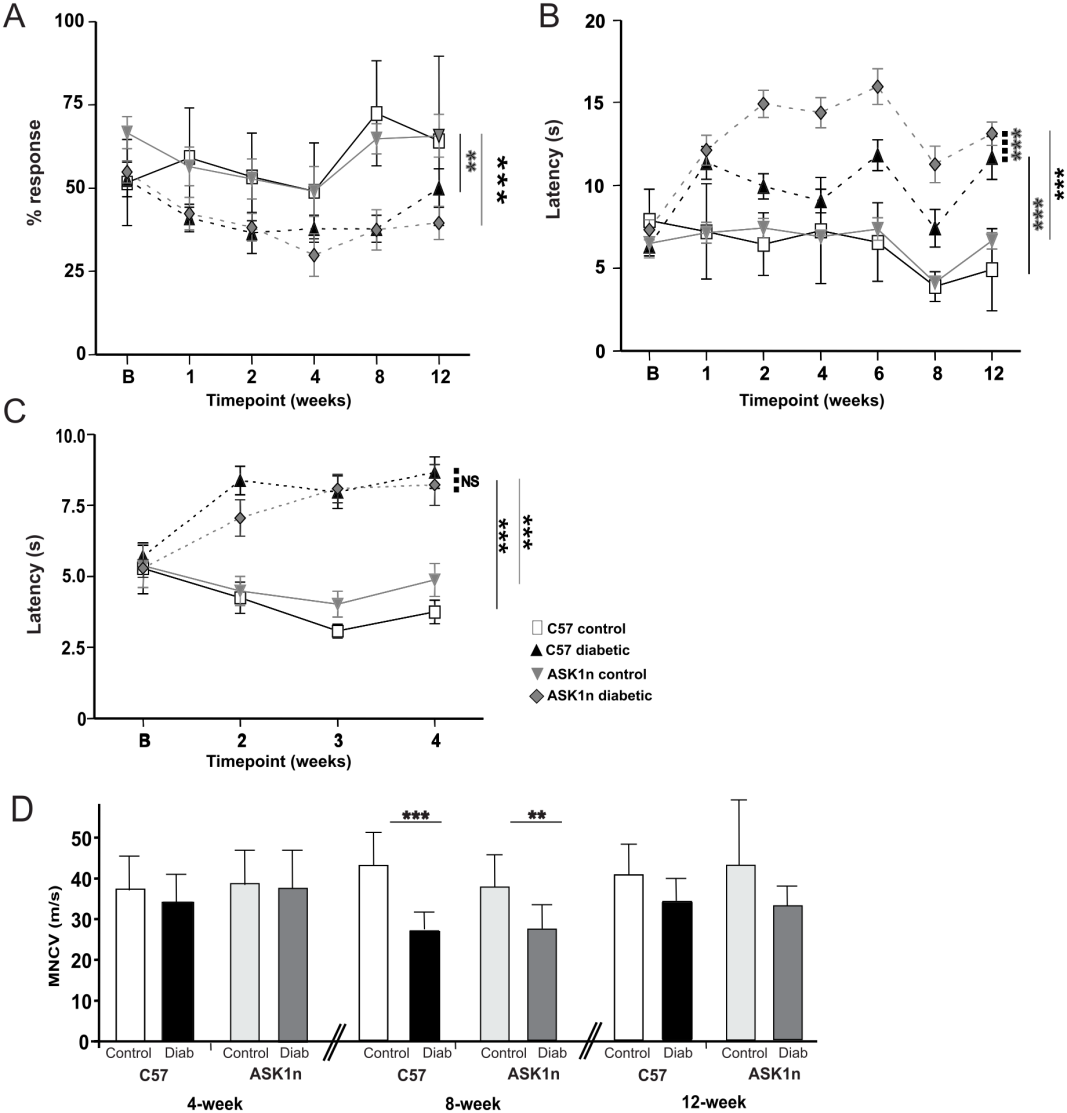
The absence of functional ASK1 does not ameliorate diabetes-associated sensory loss or nerve conduction velocity deficits. (A) Withdrawal responses to hindpaw mechanical stimulation (a 2 g Von Frey filament) touched to the hindpaw show that ASK1n mice develop sensory hypoalgesia in diabetes, similar to C57 mice. (B) The mean time taken for withdrawal of hindpaws from an infrared heat source is increased as a result of diabetes. There was exacerbation of this thermal hypoalgesia in one cohort of diabetic ASK1n mice, but was not repeated in two subsequent trials (one shown in C; NS not significant; **p<0.01; ***p<0.001 in a 2-way ANOVA on area under curve, with Bonferroni *post hoc* tests. Mean values ± SEM are displayed (*n* = 9–13)). (D) Motor nerve conduction velocities (MNCV) deficits were measured in both C57 and ASK1n mice from 8 weeks of diabetes compared with controls; p<0.001 at 8 weeks and p<0.05 at 12 weeks overall in a 2-way ANOVA, **p<0.01, ***p<0.001 in Bonferroni *post hoc* tests (*n* = 9–11). Note, there were no significant differences in near-nerve temperature in any group apart from the 8-week C57 cohort (control 33.9±0.6°C, diabetic 33.0±0.9°C, p<0.05; 2-way ANOVA, with Bonferroni *post hoc test*).

Control C57 and ASK1n mice maintained a relatively consistent withdrawal response to a 2 g Von Frey filament over 12-weeks, with no difference observed between genotypes (Fig. 7A). Diabetic mice were less sensitive to touch stimuli than control mice, and showed a reduced response to mechanical stimuli by 1 week of diabetes, which persisted over the 12-week timecourse. This was typified at 8 weeks of diabetes, with a 36±16% (diabetic C57) and 34±22% (diabetic ASK1n) response rate to the 2 g filament, compared with a response rate of 72±16% (C57) and 65±14% (ASK1n) of control mice. This equated to a significant reduction in mechanical sensitivity in diabetic mice (p<0.001, overall in a 2-way ANOVA at 8 weeks), with no significant difference observed between genotypes.

The thermal sensitivity thresholds of control mice were similarly consistent over the 12-week timecourse (Fig. 7B). From 1 week onwards, diabetic mice were less sensitive to thermal stimuli (p<0.001 overall in a 2-way ANOVA at 8 weeks). For example at 8 weeks of diabetes, C57 control mice and ASK1n mice took 3.9 s±0.9 and 4.1 s±0.8, respectively, to remove their paw from the heat source. Diabetic C57 mice took 7.8 s±4.4 and diabetic ASK1n mice took 11.0 s±3.8 (Fig. 7B). In this cohort of animals, diabetic ASK1n mice were consistently more insensitive to heat than their diabetic C57 counterparts, a deficit which was evident from week 2 and continued over the next 6 weeks of testing (p<0.001 using area under the curve values across the whole timecourse). Two further behavioural studies were conducted over 4 weeks (one is shown in Fig. 7C, Table 1F), to ascertain whether the exacerbation of thermal hypoalgesia in diabetic ASK1n mice was repeated. However, in these two studies, reduced thermal thresholds appeared equivalent between the genotypes. No significant diabetes-associated loss in intraepidermal nerve fibre (IENF) density was detected at 12 weeks and IENF density was equivalent between genotypes (Figure S1). In addition, regenerative potential was unaffected by non-functional ASK1, with neurotrophin-stimulated neurite outgrowth from dissociated adult mouse sensory neurons equivalent between genotypes (Figure S2).

An often used end-point of diabetic neuropathy in preclinical and clinical trials is a reduction in motor nerve conduction velocity (MNCV, Fig. 7D). After 8 weeks of diabetes, MNCV was significantly reduced in both C57 (control: 43 m/s±8.1 vs diabetic: 26 m/s±4.2, p<0.001) and ASK1n diabetic mice (control: 38 m/s±7.9 vs diabetic: 27 m/s±5.8, p<0.01). An overall significant reduction (p<0.05) was also measured in the 12-week diabetic animals (C57 34 m/s±5.4; ASK1n 33 m/s±4.7) compared with their controls (C57 40 m/s±7.5, ASK1n 43 m/s±16.0), but not in *post hoc* tests. There was no difference in MNCV between the genotypes at any timepoint tested.

Therefore, lack of functioning ASK1 does not prevent nor ameliorate diabetes-associated mechanical or thermal hypoalgesia or nerve conduction velocity deficits.

## Discussion

Increased p38 MAP kinase activation has been implicated in the etiology of a variety of behavioural and electrophysiological indices of diabetic neuropathy [25]. It has been described in both hyperalgesic [16], [17], [26] and hypoalgesic [15] experimental models, with an increase in total p38 observed in sural nerve from patients with type 1 and type 2 diabetes [13]. Furthermore, daily administration of a p38 inhibitor to hyperalgesic STZ-diabetic rats normalises mechanical thresholds to baseline values [16] and also prevents nerve conduction velocity deficits in STZ rats [17].

As a common upstream activator of both p38 and JNK [6], we hypothesised that activation of ASK1 may contribute to the nerve dysfunction observed in diabetic neuropathy. In this study, we show that ASK1 mRNA and protein is constitutively expressed in sensory neurons of lumbar DRG. We detected the ASK1 transcript in the sciatic nerve, DRG and spinal cord, in addition to other tissues previously shown to express ASK1 [1], [19]. The universal ASK1 expression is not surprising given that its primary function of inducing apoptosis is a somewhat ubiquitous one. ASK1-ir was observed in sensory neurons within the DRG, in a range of sizes of neuronal profiles, suggesting that expression of ASK1 is not restricted to a single population of sensory neurons. The expression of neurons with high ASK1-ir may reflect constitutive ‘injury/remodeling’ levels such as that seen with basal expression levels of ATF3, an injury-induced member of the activating transcription factor/cAMP-responsive element binding protein family [27]. In diabetes, nerves are exposed to constantly fluctuating blood sugar levels resulting in, amongst other effects, increased nitrosative-oxidative stress [28], [29], therefore we hypothesized that the expression and regulation of ASK1 [10] may be altered in the sensory nervous system in diabetes. However, we found that levels of ASK1 mRNA and protein did not change in the DRG, spinal cord, or sciatic nerve after either 4 or 12 weeks of diabetes. Thus, diabetes *per se* does not change the expression or levels of ASK1 in these tissues at these time points.

However, our study did reveal a significant increase in ASK2 mRNA in the spinal cord at 4 weeks of diabetes. ASK2 is a MAP 3 kinase related to ASK1 [2] and is able to form a heterodimer with ASK1, promoting its activation [11]. ASK2 is highly expressed in lung, skin and the gastrointestinal tract, all tissues exposed to highly fluctuating environments [2], [30]. ASK2 has been shown to facilitate oxidative stress-mediated apoptosis [11], although in different models its role is more complex and maybe linked to its relative levels with respect to ASK1 [5], [30], [31]. Unfortunately, lack of commercial antibodies prevented us from further characterising ASK2 protein levels, but we suggest that changes in ASK2 levels could alter ASK1 regulation and levels of oxidative stress in diabetes.

A number of protective phenotypes have previously been reported in ASK1 knock-out mice. For example, attenuation of 14-3-3 (a negative regulator of ASK1) in diabetic mice worsened cardiomyopathy, suggesting that the up regulation of 14-3-3 (and subsequent prevention of ASK1 activation) would be beneficial in lessening ventricular dysfunction associated with diabetic cardiomyopathy [8]. Some of these protective phenotypes may in fact be due to the loss of not just ASK1, but also ASK2. ASK1 is required for ASK2 stability, and without which, ASK2 is degraded by the ubiquitin-proteosome system [11]. Similarly, little is known about the expression and function of the recently described ASK3 [3], but it is possible that its stability may also be dependent on binding ASK1. Indeed, ASK1 possesses numerous other important scaffolding and non-enzymatic functions, therefore instead of a full ASK1 knockout, we utilized a transgenic ASK1n mouse. This was to model the action of a small-molecule ASK1 inhibitor, which would block the kinase activity, but not affect its other functions. Akita mice, which exhibit pancreatic beta cell death, are delayed in their onset of diabetes when ASK1 is knocked out [32]. However, we were able to induce experimental diabetes in transgenic ASK1n mice using streptozotocin, but saw no functional protective effects.

Similar to other groups using a single high-dose of STZ [33]–[35], we found mechanical and thermal sensitivity thresholds to be significantly reduced in diabetic mice compared with controls, and lack of functioning ASK1 did not protect against this. The mechanism of early-stage diabetic-induced hypoalgesia is not known, and often appears before any loss in intraepidermal nerve fibre innervations (Beiswenger et al, 2008), this suggests at a mechanism involving changes to neurotransmitter, cytokines or growth factors and/or their associated receptors. Whilst we did find an increase in levels of phosphorylated-p38 relative to ERK in sciatic nerve from diabetic mice, we found no functional protective effects in ASK1n mice against diabetes-associated thermal and mechanical hypoalgesia or the nerve conduction velocity deficits. This is in contrast to findings presented in abstract form which describe reduced pain responses in ASK1n mice with a Freunds complete adjuvant-induced inflammation ([20], reviewed in [36]).

Perhaps targeting p38 activation in hypoalgesia through an alternate pathway may prove beneficial. For example, NF-κB activation in the DRG was reduced in TNF-α knock-out mice protected from STZ-diabetes-associated reductions in motor NCV and thermal hypoalgesia [37]. Alternatively, targeting both ASK1 and ASK2 isoforms, to remove any compensatory effects might prove effective in alleviating symptoms of diabetic neuropathy. Activation of p38 MAP kinase has been previously described in hypersensitive diabetic mice, such as db/db mice, as well as STZ-rats [28], [38], highlighting the importance of p38 to the aetiology of painful diabetic neuropathy. Therefore, targeting ASK1 kinase may still prove beneficial in hypersensitive/painful diabetic neuropathy. However, these current results suggest ASK1 as not being a suitable target to prevent or ameliorate diabetes-associated sensory loss or NCV deficits.

## Supporting Information

Figure S1 **Intraepidermal nerve fibre density in control and diabetic mice is not significantly reduced at 12 weeks.** (A–D): Representative images of PGP 9.5-ir of transverse plantar skin sections of the hind-paw of C57 (A&B) and ASK1n (C&D) control and diabetic (Diab) mice. Arrows point to examples of nerve fibres crossing into the epidermis. (E) Number of nerve fibres per mm of epidermis/dermis boundary of C57 and ASK1n control and diabetic mice. Mean values + SD are displayed (*n* = 5). Scale bar represents 50 µm.
(EPS)

Figure S2 **There is no difference in neurotrophin-mediated neurite outgrowth from sensory neurons obtained from C57 and ASK1n mice.** (A) Representative images of neurite outgrowth from dissociated sensory neurons from adult C57 and ASK1n mice, plated in either control media (no neurotrophins, A & C) or nerve growth factor (NGF 10 ng/ml, 18 hours(B & D)). (E) There is a significant increase in the numbers of sensory neurons from both genotypes that extend neurites in response to NGF compared to control media alone (D, media alone). The length of longest neurite is significantly greater in the presence of NGF and neurotrophin-3 (NT-3), but not Glial cell derived neurotrophic factor (GDNF), in neurons from both genotypes. Scale bar represents 50 µm. **p<0.01, ***p<0.001 in a 2-way ANOVA with Bonferroni *post hoc* tests (*n* = 5–6). Mean values +SD are displayed.
(EPS)
